# Lupins in the genome editing era: advances in plant cell culture, double haploid technology and genetic transformation for crop improvement

**DOI:** 10.3389/fpls.2025.1601216

**Published:** 2025-06-24

**Authors:** Krishna Mohan Pathi, Thorben Sprink

**Affiliations:** Julius Kühn Institute (JKI) – Federal Research Centre for Cultivated Plants, Genome Editing and Synthetic Biology Group, Institute for Biosafety in Plant Biotechnology, Quedlinburg, Saxony-Anhalt, Germany

**Keywords:** alkaloids, alternative plant proteins, genetic engineering, micropropagation, protoplast culture, recalcitrant species

## Abstract

The global trend towards plant-based protein sources as an alternative to animal-derived protein has surged due to health benefits, rising adoption of vegan and vegetarian lifestyles. This shift promotes sustainable agriculture by mitigating greenhouse gas emissions and safeguarding biodiversity. Among various plant-based protein sources, legumes have received considerable attention due to their high-protein content, gluten-free nature and nitrogen-fixing capacity, making them indispensable in crop rotation systems. Within the legume family, lupins are gaining global attention for their exceptional nutritional profile and bioactive compounds with promising health benefits. Although lupins offer significant nutritional benefits, challenges such as biotic and abiotic stresses and anti-nutritional factors persist. Addressing these challenges demands advanced breeding techniques capable of mitigating these issues without compromising desirable traits. Genome editing holds promise for enhancing crop traits, including improved nutritional value and resistance to environmental stresses. The availability of complete genome sequences for lupin species provides a foundation for genome editing and accelerated breeding. However, genome editing requires reproducible plant cell culture and transformation protocols. Nonetheless, legumes exhibit a high degree of recalcitrance to *in vitro* regeneration and genetic transformation, the underlying mechanisms of which remain largely unknown. This review provides a comprehensive examination of the current advancements, challenges and future prospects associated with plant cell culture, genetic transformation, genome editing and double haploid (DH) technologies in the context of lupin improvement. Additionally, this review briefly discusses major obstacles in conventional lupin breeding.

## 1 Introduction

Lupins (members of the *Fabaceae* family) are distinguished by their ornamental value, characterized by the production of vividly colored inflorescences (Kotecki, 2015). These plants are part of the *Lupinus* genus, which is highly diverse, encompassing over 300 species (Hughes and Eastwood, 2006; Drummond et al., 2012). Lupins are particularly noteworthy for their high-protein content, abundance of dietary fiber and low fat levels (**Table 1**). They serve as promising alternatives to processed flours in food formulations and can be used in dairy analogs such as cheese, yogurt and ice cream (Duarte et al., 2022; Pereira et al., 2022). The primary lupin species cultivated as contemporary grain crops include white lupin, yellow lupin, narrow-leafed lupin and Andean lupin (Hondelmann, 1984; Yorgancilar et al., 2009). These species exhibit distinct morphological, physiological and agronomic traits, making them valuable for various agricultural and ecological applications (Pszczółkowski et al., 2025).

**Table 1:** Nutritional, chemical and alkaloid composition of cultivated lupin species.

Category	Parameter	*L. albus*	*L. angustifolius*	*L. luteus*	*L. mutabilis*
Chemical Composition	Crude Protein (% of DM)	33–47	31–37	37–38	32–52
	Crude Fibre (% of DM)	13–16	15–17	12–15	10
	Metabolizable Energy (MJ/kg DM)	13–16	12–13	10	N/A
	Oil (%)	6–13	6–7	5–9	13–24
	Total Oligosaccharides (% of DM)	7–8	8–9	N/A	N/A
	Non-Starch Polysaccharides (%)	18	47–51	N/A	N/A
Essential Amino Acids	Lysine	4.9–5.1	4.5–5.0	4.2–4.6	5.0–7.3
	Methionine	0.6–0.7	0.6–0.7	0.6–0.7	0.4–1.4
	Cysteine	1.8–2.1	1.3–1.6	1.8–2.5	1.4–1.7
	Leucine	7.5–8.0	6.0–7.6	6.1–7.3	5.7–7.8
	Threonine	3.1–4.0	3.0–3.3	2.6–3.2	3.0–4.0
Quinolizidine Alkaloid Composition	Albine	15	ND	ND	ND
	Ammodendrine	ND	ND	ND	2
	13α-Angeloyloxylupanine	ND	ND	ND	2
	Angustifoline	ND	10	ND	1
	3-Hydroxylupanine	ND	ND	ND	12
	13-Hydroxylupanine	8	12	ND	12
	Lupanine	70	70	ND	46
	Lupinine	ND	ND	60	ND
	Multiflorine	3	ND	30	ND
	Sparteine	ND	ND	ND	16
	Tetrahydrorhombifoline	ND	ND	ND	2

The nutritional, chemical and alkaloid profiles of cultivated lupin species are summarized in **Table 1**, drawing on established data sources (Wink et al., 1995; Petterson, 1998). The data are presented as ranges or percentages, where applicable and include key metrics such as protein content, fat composition, fiber and alkaloid concentrations. Footnotes are included to clarify specific terms: DM denotes Dry Matter, "N/A" indicates Not Applicable and "ND" signifies Not Detected.

**Table 2:** Regeneration in lupin species: a comparative analysis of organogenesis, somatic embryogenesis and callus-based regeneration methods.

S. No.	Scientific Name	Accession	Explant		Callus Formation	Regeneration	Literature
Organogenesis and Callus Based Shoot Formation							
1	*L. albus*	Unknown accession		Stem tips	N/A	N/A	Ball, 1946
2	*L. hartwegii*	Lindl		Embryos from mature seed, shoot apex, hypocotyl, primary root tip	Yes	Shoot organogenesis	Lee, 1955
3	*L. hartwegii*	Lindl		Embryo-derived callus	Callus	No regeneration observed	Lee, 1955b
4	*L. angustifolius*, *L. luteus*, *L. albus*	*L. angustifolius* (cvs. Kubesa, Steb, Stevens), *L. luteus* (cvs. Palfa, Topaz, Barpine), *L. albus* (cvs. Marocco, Multolupa)		Nodal segments	Not reported	Shoot organogenesis	Schäfer-Menuhr, 1985
5	*L. texensis*	Unknown accession		Cotyledonary node	Not reported	Adventitious shoots	Upadhyaya et al., 1992
6	*L. albus*, *L. luteus*, *L. angustifolius*, *L. hispanicus*, *L. polyphyllus*	*L. albus* (cvs. Wat, Hetman), *L. luteus* (cvs. Topaz, Iryd), *L. angustifolius* (cv. Remik)		Cotyledonary node	Not reported	Shoot organogenesis	Rybczyński and Podyma, 1993
8	*L. luteus*	cv. Aurea		Hypocotyl segments	Not reported	Shoot organogenesis	Daza and Chamber, 1993
9	*L. mutabilis*	Lines LM15, LM22, LM32, LM33, LM169		Stem, leaf petioles, immature leaflets	Yes	No regeneration observed	Phoplonker and Caligari, 1993
10	*L. mutabilis, L. albus*	*L. albus* cv. Lublanc		Hypocotyl thin cell layers	Not reported	Adventitious buds	Mulin and Bellio-Spataru, 2000
11	*L. luteus*, *L. albus*, *L. angustifolius*, *L. mutabilis*	*L. luteus* (cvs. Ventus, Juno, Parys, Popiel), *L. albus* (cv. Bac, breeding line R 529-1), *L. angustifolius* (cvs. Bar, Emir), *L. mutabilis* (population No. 21756)		Axillary buds	Not reported	Shoots by organogenesis	Pniewski et al., 2002
12	*L. albus*	Unknown accession		Cotyledonary node	Not reported	Shoots by organogenesis	Aslam et al., 2020
Somatic Embryogenesis							
13	*L. albus* *L. angustifolius*, *L. luteus* *L. mutabilis*	*L. albus* - cv. BAC (B), cv. Kalina(K); *L. angustifolius* - cv. Emir. (E), cv. Mirela (M), line R-7101 (R); *L. luteus* - cv. Topaz (T),cv. Ventus (V); *L. mutabilis* - line P. Seeds of B,K, E, M	Immature embryos		Not reported	Direct somatic embryogenesis	Nadolska-Orczyk, 1992
14	*L. albus* *L. luteus* *L. angustifolius* *L. hispanicus*	*L. albus* (cvs. Wat and Hetman) *L. luteus* (cvs. Topaz and Iryd) *L. angustifolius* (cv. Emir and WTD 386) *L. hispanicus*	Immature embryos		Not reported	Direct somatic embryogenesis	Rybczyński and Podyma, 1993
Immature Embryo Cultivation							
15	*L. albus × L. luteus* *L. albus × L. angustifolius* *L. angustifolius × L. luteus* *L. luteus × L.albus* *L. luteus × L. angustifolius*	Not detected	Hybrid embryos (interspecific hybridization)		Not reported	No regeneration observed	Jaranowski, 1962
16	*L. albus* *L. angustifolius* *L. luteus* *L. mutubilis*	Not detected	Hybrid embryos (interspecific hybridization)		Not reported	Embryo germination	Williams et al., 1980
17	*L. luteus*, *L. mutabilis* *L. hartwegii*	*L.luteus*- Topaz *L. mutabilis*-BGRC 23 460 *L. hartwegii*-L 7/3,	Hybrid embryos (interspecific hybridization)		Not reported	No regeneration observed	Busmann-Loock et al., 1991
18	*L. mutabilis* × *L. hartwegii*	Not detected	Hybrid embryos (interspecific hybridization)		Not reported	Multiple shoot formation	Schäfer-Menuhr, 1988
19	*L. albus* *L. mutabilis*	Not detected	Immature embryo		Not reported	Germination	Vuillaume and Hoff, 1986
20	*L. albus* *L. angustifolius* *L. hispanicus* *L. luteus* *L. polyphyllus*	Not detected	Immature embryo and shoot tips		Callus	Shoot regeneration and organogenesis	Podyma et al., 1988

**Table 2** provides a systematic comparison of regeneration efficiency in lupin species across three primary methodologies: organogenesis, somatic embryogenesis and callus-based regeneration. The table delineates key parameters such as accessions and explant types, Footnotes are included for clarity: "N/A" denotes Not Applicable and "cv." refers to cultivar.

**Table 3:** Application of double haploid (dh) technology in lupin species.

S. No.	Scientific Name	Accession	Explant	Induction Treatment	Callus Formation	Plant Regeneration	Reference
1	*L. polyphyllus*	Not specified	Anthers	Not specified	Yes	No regeneration observed	Sator et al., 1983
2	*L. polyphyllus*, *L. hartwegii*, *L. angustifolius*, *L. luteus*	*L. angustifolius* Accession Kubesa, *L. luteus* Accessions Barpine, Palfa, Topaz	Anthers	Not specified	Yes	Only from *L. polyphyllus*	Sator et al., 1985
3	*L. albus*	Not specified	Anthers and microspores	Not specified	Yes (Embryo-like structures)	No regeneration observed	Ormerod and Caligari, 1994
4	*L. albus*, *L. angustifolius*, *L. luteus*	*L. albus* cv. Kiev Mutant, *L. angustifolius* cv. Marri, cv. Chittick, *L. luteus* cv. Wodjil	Microspores	4°C, 32°C	Multicellular pro-embryos	No regeneration observed	Bayliss et al., 2004
5	*L. angustifolius*, *L. albus*, *L. luteus*	*L. angustifolius* cv. Polonez, cv. Sonet, *L. albus* cv. Katon, cv. Wat, *L. luteus* cv. Legat, cv. Juno	Anthers	4°C, 32°C	Yes	No regeneration observed	Skrzypek et al., 2008
6	*L. angustifolius*	cv. Emir, cv. Graf	Anthers and microspores	4°C	Yes	No regeneration observed	Kozak et al., 2012

**Table 3** provides a detailed overview of the application of Double Haploid technology in lupin species, highlighting critical parameters such as accessions, explant types, induction treatments, callus formation and plant regeneration efficiency. Footnotes are included for clarity: "N/A" denotes Not Applicable and "cv." refers to cultivar.

White lupin (*Lupinus albus*, 2n = 50, genome size: 653 Mb (Hufnagel et al., 2020)) is characterized by its high-protein content, palmate leaves and spirally arranged dorsal flowers, which are predominantly white but may also appear in blue or pink hues. A defining physiological characteristic of this species is its capacity to form cluster roots specialized structures that exude carboxylates, facilitating phosphate mobilization and improving soil nutrient availability (Petterson, 2016; Abraham et al., 2019; Alkemade et al., 2021). The cultivation of *L. albus* began approximately 4,000 years ago (Wolko et al., 2011). Yellow lupin (*L. luteus*, 2n = 52, estimated genome size: 1,024.49 Mb (Lichtin et al., 2020)) is an annual species notable for its nitrogen-fixing ability and dense clusters of bright yellow flowers. Its palmate leaves and adaptability contribute to its attractiveness for pollinators and ornamental use (Kotecki et al., 2020; Martinez-Hernandez et al., 2024). Narrow-leafed lupin (*L. angustifolius*, 2n = 40, genome size: 975 Mb (Garg et al., 2022)), commonly known as blue lupin, this species has narrow leaves and blue-violet flowers. Breeding efforts have successfully reduced its alkaloid content, resulting in the development of “sweet” cultivars that are now extensively cultivated for human and animal consumption (Pszczółkowski et al., 2025). Andean lupin (*L. mutabilis*, 2n = 48, genome size: 620 Mb (Pancaldi et al., 2024)), commonly known as tarwi, is a perennial species with multi-colored flowers that transition from white with yellow wings to dark purple. Its nutritional value and adaptability are promising, though it requires careful pest and disease management (Guilengue et al., 2020; Pszczółkowski et al., 2025).

Lupins are unique among protein crops, with seed protein content reaching up to 44%, comparable to soybeans, making them one of the highest-protein plant species (Lucas et al., 2015). Moreover, lupins are generally more tolerant to various abiotic stresses compared to other legumes and they hold significant potential for the restoration of degraded or nutrient-poor soils (Lucas et al., 2015).

Lupins offers health benefits such as improved bowel function, cholesterol reduction and blood glucose regulation (Van De Noort, 2017). Despite their historical use dating back to ancient Egypt and pre-Incan South America, lupins remain an underutilized legume in modern diets (Lucas et al., 2015). Europe’s heavy reliance on soybean imports, governed by trade agreements and quality standards, does not satisfy expectations of European citizens (Lucas et al., 2015). Thereby, Native European lupins, such as *L. albus*, *L. luteus* and *L. angustifolius* offer promising alternatives to soybeans due to their high-quality protein, potential health benefits and suitability for sustainable production (Lucas et al., 2015). However, lupin cultivation in Europe is still insufficient to ensure a consistent supply to the food industry, which must innovate to create appealing lupin-based protein-rich products (Lucas et al., 2015). Despite their agronomic and nutritional advantages, lupins remain underutilized due to significant breeding challenges (Pszczółkowski et al., 2025). Some of these constraints are discussed under the subheading *Challenges in lupin breeding in this review.*

Lupins exhibit two distinct phenotypes: bitter and sweet defined primarily by their alkaloid composition, which influences both edibility and sensory attributes. These alkaloids render the seeds unpalatable and pose neurotoxic risks to humans and animals (Maknickienė and Asakaviciute, 2008). To address these safety concerns, regulatory authorities in New Zealand, Australia, the United Kingdom and France have set a maximum allowable alkaloid limit of 200 mg/kg in lupin-based food products (Resta et al., 2008).

Bitter lupins synthesize a diverse array (**Table 1**) of nitrogen-containing secondary metabolites known as quinolizidine alkaloids (QAs) (Wink et al., 1995; Petterson, 1998). These alkaloids serve as chemical defenses against herbivores and exhibiting antimicrobial activity (Erdemoglu et al., 2007; Mancinotti et al., 2023). QAs are biosynthesized from the amino acid L-lysine through a series of enzymatic steps involving decarboxylation, oxidation and cyclization (Mancinotti et al., 2025). Recent studies have proposed the involvement of six to nine enzymes in this pathway, although the complete sequence of reactions and all participating enzymes have yet to be fully elucidated (Mancinotti et al., 2022).

Advancements in metabolic engineering have enabled the manipulation of QA biosynthesis in lupins (Ramírez-Betancourt et al., 2021). For instance, *L. angustifolius* has been engineered to accumulate elevated levels of sparteine, a QA of industrial relevance due to its role in asymmetric synthesis (Mancinotti et al., 2025). Manipulation of QA biosynthesis remains a key objective in lupin biotechnology, aiming to further reduce anti-nutritional compounds in edible seeds, enhance plant defense mechanisms and facilitate the production of valuable alkaloids for pharmaceutical applications (Osorio and Till, 2021; Mancinotti et al., 2022, 2023, 2025). Current strategies include CRISPR (Clustered Regularly Interspaced Short Palindromic Repeats) -based genome editing, RNA interference for targeted gene suppression and heterologous pathway reconstruction. However, the effectiveness of these approaches is constrained by the limited amenability of lupins to tissue culture and genetic transformation.

### 1.1 Challenges in lupin breeding

The primary objective of contemporary lupin breeding programs is to develop cultivars with enhanced agronomic traits, such as reduced alkaloid content, enhanced disease resistance, greater adaptability to climate change and increased productivity (Pszczółkowski et al., 2025). Fungal pathogens, including anthracnose (*Colletotrichum lupini*), fusarium wilt (*Fusarium oxysporum* ssp. *lupini*) and lupin leaf fall (*Pleospora herbarum*), pose significant threats to lupin cultivation. These phytopathogens adversely affect both agricultural productivity through yield reduction and seed safety via mycotoxin accumulation (Horoszkiewicz-Janka et al., 2011; Sawicka and Pszczółkowski, 2014; Kotecki et al., 2020). Consequently, breeding programs must prioritize the development of lupin cultivars exhibiting improved resistance against both major pathogens and additional biotic constraints, including gray mold, viral diseases, aphid infestations and competitive weed pressure (Sawicka and Pszczółkowski, 2014).

Yield improvement remains another critical area of research in lupin breeding. Recent efforts have leveraged genomic tools to identify yield-associated genes and integrate them into elite lupin varieties (Mavromatis et al., 2023). Climate change further complicates breeding, necessitating resilient varieties adaptable to shifting environmental conditions. Interspecific hybridization within lupins is challenging, as evidenced by limited success in crossing species such as *L. albus* and *L. mutabilis* (Sawicka-Sienkiewicz et al., 2006).

Lupin breeding faces significant challenges in disease resistance, yield enhancement and climate resilience (Pszczółkowski et al., 2025). Scientists are exploring various methods to develop improved lupin varieties, with new genomic techniques revolutionizing plant biotechnology. These advanced methodologies provide numerous advantages, notably their precision and efficiency in the targeted introduction of specific traits into the plant genome. Genome editing, a targeted approach, allows for precise alterations in the plant’s DNA, enabling the enhancement of desirable traits (Abdul Aziz et al., 2022; Yıldırım et al., 2023). The availability of complete genome sequences for lupin species provides a solid foundation for genome-editing research and accelerated breeding efforts (Hufnagel et al., 2020; Wang et al., 2021; Garg et al., 2022).

The limited application of genome editing in certain legume crops underscores the need for further research and innovation to fully leverage the potential of lupins and other legumes for sustainable protein production (Nivya and Shah, 2023). The development of new lupin varieties is crucial to ensuring adaptability and nutritional quality, taking into consideration factors such as cultivation conditions and climate variability. Plant cell culture is integral to both conventional and contemporary breeding methodologies, serving a pivotal function in the advancement of crop improvement strategies (Pathi and Sprink, 2023). In classical breeding, it facilitates the rapid production of double haploid plants, speeding up the breeding process by enabling the identification of desirable traits and more efficient selection of improved plant varieties (Murovec and Bohanec, 2012). In modern breeding, plant cell culture serves as a powerful tool for genetic manipulation and the propagation of desired traits. Within the framework of genome editing, it allows for precise modifications at the cellular level, leading to targeted improvements in plant DNA (Loyola-Vargas and Avilez-Montalvo, 2018). Additionally, plant cell culture supports the mass production of genetically modified plants, ensuring a sufficient number of plants with enhanced genetic traits is being generated (Kowalczyk et al., 2022).

To the best of our knowledge, this is the most comprehensive review to date on the *in vitro* and biotechnological aspects of lupins. It provides an in-depth examination of key areas including plant tissue culture, genetic transformation, protoplast technology, double haploid production and genetic engineering, while also highlighting existing challenges and future prospects.

## 2 Lupin cell/tissue culture

Biotechnological approaches such as *in vitro* mutagenesis, protoplast culture-mediated somatic hybridization and genetic transformation support advances in lupin breeding. These methods rely on optimized protocols for plant cell/tissue culture. However, lupin explants exhibit poor *in vitro* response.

**Figure 1:**
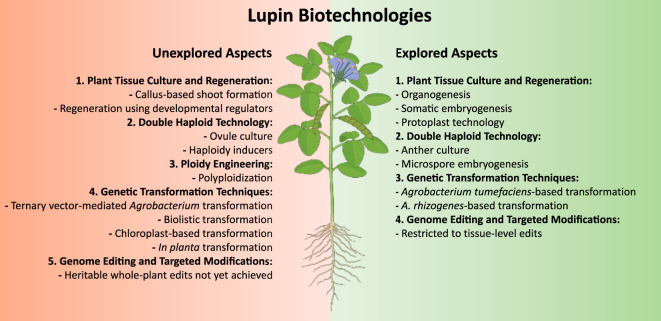
Schematic representation of explored and unexplored aspects in lupin biotechnology. **Figure 1** presents a detailed schematic representation of the current research landscape in lupin biotechnology, systematically categorizing both explored and unexplored domains. Explored areas are depicted in green, while unexplored aspects are highlighted in red, providing a visually intuitive distinction between established knowledge and potential avenues for future investigation. This scheme was generated using BioRender.

Despite these challenges, the totipotency of plant cells enables several promising biotechnological applications. While a few successful reports exist, comprehensive efforts in lupin *in vitro* propagation remain limited (**Figure 1**). The following sections review the current achievements, challenges and future prospects in this area.

### 2.1 Organogenesis

Numerous studies have investigated *in vitro* organogenesis in lupins, consistently identifying explant source as a critical determinant of morphogenic competence. A diverse range of explants has been evaluated, such as apical meristems (Ball, 1946; Lee, 1955a), shoot tips (Rybczynski and Podyma, 1993; Pigeaire et al., 1997), axillary buds (Schäfer-Menuhr, 1985; Pniewski et al., 2002), nodal regions (Mulin and Bellio-Spataru, 2000), cotyledonary nodes (Upadhyaya et al., 1992; Aslam et al., 2020) and hypocotyl-derived explants (Daza and Chamber, 1993). Among these, apical meristems, axillary buds, hypocotyl-derived explants and cotyledonary nodes consistently exhibit the highest capacity for multiple shoot induction. High shoot multiplication rates have been reported in *L. hispanicus* (Rybczynski and Podyma, 1993), *L. mutabilis* (Pniewski et al., 2002), *L. texensis* (Upadhyaya et al., 1992), *L. albus* (Aslam et al., 2020) and *L. luteus* (Daza and Chamber, 1993).

Explant age further modulates regenerative outcomes. Younger tissues, particularly cotyledonary nodes, generally exhibit superior morphogenic responses, as demonstrated in *L. albus* (Aslam et al., 2020). However, exceptions to this trend highlight the complexity of regeneration biology. For instance, *L. albus* explants derived from 5-day-old seedlings showed no response under *in vitro* conditions (Rybczynski and Podyma, 1993). In contrast, the regenerative potential of apical meristems appears independent of age, with successful organogenesis observed from 30-day-old seedlings of *L. albus* (Ball, 1946) and water-imbibed mature embryos of *L. hartwegii* (Lee, 1955a). These observations underscore the role of genotype-specific physiological status in determining morphogenic competence.

Most regeneration protocols in lupins utilize Murashige and Skoog (MS) or Gamborg B5 media, whereas early studies employed Robins formulation (Ball, 1946; Lee, 1955a). In *L. albus*, high-frequency regeneration from half cotyledonary node explants was achieved using a low-nutrient MS medium, with the inclusion of activated charcoal significantly enhancing shoot elongation and reducing tissue browning. Among the carbohydrate sources tested, sucrose led to the highest shoot regeneration frequency, particularly in half cotyledonary node explants (Aslam et al., 2020).

Plant growth regulator (PGR) combinations have a decisive impact on regeneration outcomes. Among cytokinins, benzyladenine (BA), kinetin and 2-isopentenyladenine (2iP) are commonly utilized, with BA and kinetin being particularly effective for multiple shoot induction (Upadhyaya et al., 1992; Daza and Chamber, 1993; Mulin and Bellio-Spataru, 2000; Aslam et al., 2020). Notably, BA in combination with Naphthaleneacetic Acid (NAA) significantly enhanced shoot regeneration in *L. angustifolius* (Pigeaire et al., 1997).

Considerable progress has been made in shoot induction. However, rooting remains a major bottleneck. Root formation often requires a reduction in basal medium strength and the application of auxins (Schäfer-Menuhr, 1985; Pniewski et al., 2002; Aslam et al., 2020). In *L. albus*, a rooting frequency of up to 80% was achieved within 28 days on low-strength MS medium supplemented with B5 vitamins and auxins (Aslam et al., 2020). However, prolonged *in vitro* cultivation was found to reduce rooting efficiency (Pniewski et al., 2002). In cases where shoots do not produce roots, plant establishment has been successfully achieved by grafting regenerated shoots onto decapitated seedlings (Pniewski et al., 2002), underscoring the ongoing challenges in developing robust root systems for regenerated plants.

### 2.2 Callus based shoot formation

Callus-mediated shoot regeneration in lupins remains largely unsuccessful. Earlier studies were instrumental in identifying responsive explants and showed callus formation from *L. hartwegii* shoot apices (Lee, 1955b) and various explants of *L. mutabilis* (Phoplonker and Caligari, 1993), but none of these studies achieved shoot regeneration, highlighting persistent challenges in callus-based shoot formation in lupins

### 2.3 Somatic embryogenesis

Somatic embryogenesis offers significant advantages for plant regeneration (Pathi et al., 2013; Martínez and Corredoira, 2024), yet its application in lupins remains largely underexplored. Only a limited number of studies have reported species-specific responses, consistently identifying immature cotyledons as the most responsive explant and Gamborg B5 as the optimal basal medium (Nadolska-Orczyk, 1992; Rybczyński and Podyma, 1993). Early work by Nadolska-Orczyk (1992) demonstrated successful somatic embryo induction in *L. angustifolius*, *L. albus* and *L. mutabilis*, whereas *L. luteus* failed to respond. However, despite successful induction, this protocol did not yield fully regenerated plants, highlighting the problem of poor shoot conversion. Building on this foundation, Rybczyński and Podyma (1993) achieved complete plant regeneration in *L. albus* by optimizing the hormonal regime and incorporating coconut water into the culture medium. While both protocols proved effective for *L. albus*, their limited success in other *Lupinus* species underscores the challenge of developing broadly applicable somatic embryogenesis protocols. Further research is needed to refine these approaches, overcome species-specific response and enhance shoot-to-plant conversion efficiency.

### 2.4 Immature embryo culture

Interspecific hybridization in lupins has long been constrained by pronounced cross-incompatibilities, limiting its utility in expanding genetic diversity within breeding programs (Jaranowski, 1962; Williams et al., 1980). In this context, embryo rescue techniques have emerged as a pivotal strategy to overcome post-zygotic barriers and facilitate the recovery of viable hybrid progeny (Vuillaume and Hoff, 1986; Bridgen, 1994). Several embryo rescue systems have been established, including agar medium culture (Schafer-Menuhr et al., 1988), paper bridges over liquid medium (Vuillaume and Hoff, 1986) and liquid-over-agar methods (Podyma et al., 1988). These technical advancements have identified critical developmental thresholds such as a minimum embryo size of ≥1 millimeter for successful culture and demonstrated the efficacy of coconut milk supplementation in supporting the development of early heart-stage embryos, enabling successful embryo rescue in *L. albus* and *L. mutabilis* (Podyma et al., 1988).

### 2.5 Double haploid technology

Double haploid technology is highly valuable for fundamental research and plant breeding, accelerating genetic improvement and trait selection. The advantages of DH technology in classical and new breeding methods were reviewed in a previous article (Pathi and Sprink, 2023). However, the advancement of double haploid protocols for plant improvement in *Fabaceae* has progressed slowly compared to other plant families (Croser et al., 2006; Skrzypkowski and Kiełkowska, 2024).

Research on lupin haploid plants production has demonstrated the potential for microspore-derived embryogenesis, with key studies confirming that isolated microspores of *L. albus*, *L. angustifolius* and *L. luteus* can form multicellular pro-embryos under optimized culture conditions (Ormerod and Caligari, 1994; Bayliss et al., 2004). Refinements in donor plant selection such as bud size (5–6 millimeter) and anther color have improved embryogenic responses (Skrzypek et al., 2008; Kozak et al., 2012). Skrzypek et al. (2008) reported an embryogenic response in lupin anther cultures without the need for inflorescence pre-treatment, a finding that is atypical among legume species. Early work by Sator et al. (1983); Sator (1985) demonstrated the feasibility of anther culture in *L. polyphyllus*, achieving diploid regeneration, although not true double haploids.

Despite these advancements, lupin androgenesis faces persistent challenges. A major bottleneck is the exine barrier, which restricts pro-embryo development (Bayliss et al., 2004). Unlike in model species where exine rupture occurs naturally (Daghma, 2011; Siemons et al., 2025), lupin microspores may require mechanical or enzymatic assistance, a factor that remains underexplored in current protocols. Additionally, species-specific response is evident: while *L. polyphyllus* regenerates diploid plants (Sator, 1985), other species such as *L. luteus* and *L. angustifolius* produce callus but fail to regenerate shoots. Overcoming species-specific barriers, optimizing stress pre-treatments, employing haploidy inducers and integrating insights from model systems will be critical for advancing double haploid (DH) technology in lupins.

### 2.6 Protoplast technology

Protoplasts, defined as plant cells devoid of a cell wall. The isolation and cultivation of protoplasts present numerous benefits, including opportunities for genetic manipulation, studies on hybridization, investigation into cell physiology, regeneration from a single cell and manipulations at the single-cell level. These advantages have been extensively reviewed in our previous report (Pathi and Sprink, 2023).

In recent decades, significant progress has been made in protoplast isolation and culture systems in *Lupinus* species, establishing a foundation for their use in developmental biology and biotechnology. Early work by Schäfer-Menuhr (1987; 1988; 1989) developed high-yield, high-purity protocols for mesophyll protoplast isolation in *L. angustifolius*, *L. polyphyllus* and their hybrids. Subsequent studies further identified optimal explant sources for reliable protoplast isolation. Cotyledons from *in vitro*-grown seedlings of *L. albus* and mesophyll tissues from mature leaves of *L. angustifolius* and *L. polyphyllus* have consistently produced the highest yields of viable protoplasts, reflecting the favorable cellular architecture and developmental plasticity of these tissues (Schäfer-Menuhr, 1987; Schäfer-Menuhr and Stuermer, 1987; Schäfer-Menuhr, 1988; 1989; Babaoğlu, 2000; Sinha et al., 2003; Sinha and Caligari, 2004). Despite these advances, species- and genotype-specific variability continues to hinder protocol standardization, reflecting the biological heterogeneity within the genus (Beşer and Wetten, 1996; Sinha and Caligari, 2004).

In parallel, the development of synthetic culture media initially AS 19 and later K8p (Kao and Michayluk medium) was instrumental in promoting protoplast-derived callus formation and initiating cell division and morphogenesis (Schäfer-Menuhr, 1987; 1988; 1989). Yet, despite the early formation of morphogenic structures, the regeneration of complete plants from lupin protoplasts remains elusive. Most studies terminate at the callus stage, reflecting incomplete organogenic or embryogenic competence and an ongoing inability to fully exploit the totipotent potential of protoplasts (Babaoğlu, 2000; Sinha and Caligari, 2004, 2005).

To enhance culture responsiveness, Sinha et al. (2003); Sinha and Caligari (2004); Sinha and Caligari (2005) refined multiple parameters in *L. albus* cultures, including enzyme composition, osmotic potential and pH, substantially improving protoplast viability and division rates. Despite these refinements, the developmental trajectory remains unstable. Lupin protoplasts display hypersensitivity to minor fluctuations in culture conditions, such as osmolarity and pH, which can severely compromise both cell viability and morphogenic progression (Babaoğlu, 2000; Sinha and Caligari, 2005).

Furthermore, technical innovations most notably droplet plating on Nunclon surfaces have improved protoplast elongation and mitotic activity, offering enhanced physical environments for single-cell culture (Sinha and Caligari, 2005). Nevertheless, other approaches intended to improve morphogenesis, including the use of embedding matrices such as alginate and filter paper, as well as nurse and suspension cultures, have often yielded poor outcomes (Babaoğlu, 2000; Sinha and Caligari, 2005; Wiszniewska and Pindel, 2009). This suggests that while physical handling techniques have advanced, the cellular microenvironment remains suboptimal for consistent regeneration.

In addition to their regenerative potential, lupin protoplasts have served as valuable models for physiological studies. For instance, Zhang et al. (2004) utilized root-derived protoplasts from *L. albus* to explore citrate efflux mechanisms under phosphorus deficiency, demonstrating the versatility of protoplast systems in stress physiology.

Despite notable advances in protoplast isolation and culture optimization, efficient whole-plant regeneration from isolated protoplasts remains a pivotal challenge. Addressing this limitation is essential for fully harnessing protoplast technologies in next-generation lupin breeding and genome editing initiatives. Although approaches based on somatic hybridization, discussed in the following section, have explored protoplast fusion strategies, success in achieving complete shoot regeneration has remained limited.

#### 2.6.1 Somatic hybridization

Early studies in lupins have highlighted the potential of protoplast fusion as a strategy for interspecific genetic manipulation and plant regeneration. A seminal contribution by Schäfer-Menuhr (1989) demonstrated, for the first time, that mesophyll protoplasts derived from *L. mutabilis × L. hartwegii* hybrids could be induced to form calli and regenerate shoots under optimized culture conditions. The regenerated shoots displayed morphological similarity to the parental genotype. These findings established a foundational proof-of-concept for protoplast-based regeneration in lupins. However, the absence of molecular characterization in this study left the hybrid nature and genetic stability of the regenerants unconfirmed.

Building on this work, Sonntag et al. (2009) successfully employed electrofusion of protoplasts from *L. angustifolius* and *L. subcarnosus* to generate somatic hybrid calli capable of shoot regeneration. Notably, no shoot development was observed in colonies derived from the parental protoplasts alone. Molecular marker analyses confirmed the hybrid identity of the regenerants, implicating heterotic or synergistic genetic interactions as key drivers of morphogenesis. While this study marked a significant advancement in lupin protoplast technology and genetic improvement, the reproducibility of these outcomes across other *Lupinus* species remains to be established.

**Table 4:** Application of protoplast technology in lupin species.

S. No.	Scientific Name	Accession	Tissue Source	Callus Formation	Regeneration	Literature
1	*L. angustifolius*	cv. Kubesa	Mesophyll protoplasts	Yes	No regeneration observed	Schäfer-Menuhr, 1987
2	*L. angustifolius*	cv. Kubesa	Mesophyll protoplasts	Yes	No regeneration observed	Schäfer-Menuhr and Stuermer, 1987
3	*L. polyphyllus*	Not detected	Suspension cultures	Yes	No regeneration observed	Schäfer-Menuhr, 1988
4	*L. albus*, *L. luteus*, *L. angustifolius*, *L. mutabilis*	Not detected	Leaves	Not reported	No regeneration observed	Beşer and Wetten, 1996
5	*L. albus*	CH304-70, LA132, LA156, Lucky, Lucrop	Cotyledons	Not reported	No regeneration observed	Wetten et al., 1999
6	*L. mutabilis*	cv. Potosi	Leaves, shoot tips	Not reported	No regeneration observed	Babaoğlu, 2000
7	*L. albus*	CH304/70	Cotyledons	Not reported	No regeneration observed	Sinha et al., 2003
8	*L. albus*	CH304/70	Leaves, cotyledons, hypocotyls, roots	Not reported	No regeneration observed	Sinha et al., 2003
9	*L. albus*	CH304/70	Cotyledons	Not reported	No regeneration observed	Sinha and Caligari, 2004
10	*L. albus*	Lucyanne	Cotyledons	Not reported	No regeneration observed	Sinha and Caligari, 2005
11	*L. luteus*	Parys, Taper, Mister	Hypocotyls, cotyledons, seedlings	Not reported	No regeneration observed	Wiszniewska and Pindel, 2009
12	*L. albus*	cv. Kive mutant	Roots	Not reported	No regeneration observed	Zhang et al., 2004
Somatic hybridization						
13	*L. mutabilis × L. hartwegii*	Not detected	Mesophyll protoplasts	Yes	Yes	Schäfer-Menuhr, 1989
14	*L. angustifolius, L. subcarnosus*	*L. angustifolius*: cvs. Tanjil (A8), Tallerack (A19), Probor (A23);	Young leaves	Yes	Yes	Sonntag et al., 2009
		*L. subcarnosus*: accessions 16417, 16439, 5658 (S); cvs. Vitabor (A3), Bora (A11), Arabella (A12)				

**Table 4** provides a comprehensive summary of the application of protoplast technology in lupin species, detailing critical parameters such as accessions, tissue sources for protoplast isolation, callus formation, regeneration efficiency and somatic hybridization outcomes between species. A footnote is included for clarity: "cv." refers to cultivar.

**Table 5:** Genetic transformation in lupin species.

S. No.	Scientific Name	Accession	Explant Type	Callus	Regeneration Type	Genetic Transformation Method	Candidate Gene Used	Selection/Marker Gene	Transgenicity Confirmation	Transgene Inheritance	Agronomic Trait of the Gene	Literature
1	*L. albus*, *L. polyphyllus*	Not specified	Stem segments	N/A	N/A	*Agrobacterium rhizogenes* (ATCC 31798)	N/A	N/A	Production of Agropine and Mannopine	N/A	N/A	Mugnier, 1988
2	*L. polyphyllus*, *L. hartwegii*	Not specified	3–6 old seedlings	N/A	N/A	*A. tumefaciens* (DSM-30150, B6S3, C58), *A. rhizogenes* (15834)	N/A	N/A	Southern blot	N/A	N/A	Berlin et al., 1991
3	*L. angustifolius*	cv. Warrah	Thinly sliced embryonic axes from maturing seeds	N/A	Organogenesis	*A. tumefaciens*	N/A	N/A	N/A	N/A	N/A	Molvig et al., 1997
4	*L. angustifolius*	Unicrop, Merrit	Shoot apices	N/A	Organogenesis	*A. tumefaciens*	N/A	N/A	N/A	N/A	N/A	Pigeaire et al., 1997
5	*L. mutabilis*	cv. Potosi	Apical meristem intact, extreme tip of the apical dome	N/A	Organogenesis	*A. tumefaciens* (1065)	N/A	Kanamycin	GUS assay, non-radioactive DNA-DNA hybridization	N/A	N/A	Babaoglu et al., 2000
6	*L. luteus*	Teo, Teo101, Wodjil, Popiel, Motiv 369, Juno, WDT 6174, WDT 6179	Meristem	N/A	Organogenesis	*A. tumefaciens*	N/A	N/A	N/A	N/A	N/A	Li et al., 2000
7	*L. angustifolius*	cv. Unicorp	Shoot apex	N/A	Organogenesis	*A. tumefaciens*	N/A	bar gene (glufosinate)	PCR, Southern blot	Mendelian ratio	N/A	Atkins and Smith, 2003
8	*L. luteus*	cv. Ventus	Truncated seedlings and excised hypocotyls	Callus	Callus	*A. tumefaciens*	HBV surface antigen	N/A	PCR, Southern blot	N/A	N/A	Pniewski et al., 2006
9	*L. mutabilis*	Not specified	Embryonic axes	N/A	Organogenesis	*A. tumefaciens*	Human adenosine deaminase (hADA)	β-glucuronidase (gus)	Southern blot	Mendelian segregation	N/A	Polowick et al., 2014
10	*L. angustifolius*	Mandelup	Shoot apices	N/A	Organogenesis	*A. tumefaciens*	N/A	bar gene (glufosinate)	PCR, Southern blot	Non-Mendelian inheritance	N/A	Barker et al., 2016
11	*L. angustifolius*	Mandelup	Shoot apices	N/A	Organogenesis	*A. tumefaciens*	N/A	PPT and Hygromycin	GUS assay, GFP imaging	N/A	N/A	Nguyen et al., 2016a
12	*L. angustifolius*	Mandelup	Shoot apices	N/A	Organogenesis	*A. tumefaciens*	N/A	Hygromycin	GFP imaging	N/A	N/A	Nguyen et al., 2016b
13	*L. albus*	cv. Amiga	Cotyledon	N/A	N/A	*A. rhizogenes*	Purple acid phosphatases (PAP 10, PAP12)	Bar gene	PCR	N/A	Investigate the role of PAPs in low-P availability	Xu et al., 2020
14	*L. albus*	cv. Amiga	Seedling	N/A	N/A	*A. rhizogenes*	L.albABCG29	Bar gene	PCR, GUS assay	N/A	Investigate the role of ATP-binding cassette (ABC) transporters	Aslam et al., 2021
15	*L. albus*	cv. Amiga	Root tip of the germinated seedlings	N/A	N/A	*A. rhizogenes*	LaGRAS38, LaGRAS39	Kanamycin, Hygromycin	PCR	N/A	Investigate the role of GRAS transcription factors	Aslam et al., 2023

**Table 5** provides a comprehensive overview of genetic transformation techniques applied to *Lupinus* species, delineating critical parameters such as accessions, explants used for transformation, regeneration type, genetic transformation methods, candidate genes, selection markers, transgene confirmation, inheritance patterns and associated agronomic traits. Footnotes are included for clarity: "N/A" denotes Not Applicable and "cv." refers to cultivar.

**Table 6:** Genome editing in Lupin Species.

S. No.	Scientific Name	Accession	Explant	Transformation Method	Target Gene	Agronomic Trait	Reference
1	*L. albus*	cv. Orus	Radicle	*Agrobacterium rhizogenes* (strain A4T)	LaALMT1	To investigate metal root-to-shoot translocation	Zhou et al., 2020
					Aluminium (Al)-activated malate transporter		
2	*L. albus*	cv. Orus	Radicle	*A. rhizogenes* (A4T)	LaMATE	To investigate metal root-to-shoot translocation	Zhou et al., 2021
					Multidrug and toxic compound extrusion/detoxification		
3	*L. albus*	cv. AMIGA	Radicle	*A. rhizogenes* (K599)	Putative trehalase	N/A	Zhu et al., 2023

**Table 6** provides a detailed summary of Genome editing applications in lupin species, outlining key parameters such as accessions, transformation methods, target genes and the agronomic traits associated with the genes. Footnotes are included for clarity: "N/A" denotes Not Applicable and "cv." refers to cultivar.

## 3 Genetic transformation of lupins

Plant transformation involves identifying a target gene, introducing it into plant cells and regenerating a whole plant with the expressed transgene (Chen et al., 2022). Particle bombardment and *Agrobacterium*-mediated transformation are predominantly used methods for gene transfer, though the latter has become more widely favored due to its accessibility, cost-effectiveness and ability to introduce single or low-copy transgene insertions, making it a preferred approach for plant transformation (Chen et al., 2022; Rahman et al., 2024). Stable transformation, which enables the heritable transmission of integrated genes to subsequent generations, is essential for both functional genomics and transgenic breeding applications (Rahman et al., 2024).

### 3.1 *Agrobacterium*-mediated transformation

Extensive efforts have been undertaken to establish *Agrobacterium*-mediated genetic transformation systems in lupins, progressing from early proof-of-concept experiments to more refined protocols capable of generating transgenic lines with agronomically beneficial traits. Despite these advancements, progress has been uneven across species and various technical and biological limitations continue to hinder widespread implementation. Transformation studies have predominantly relied on meristematic tissues (embryonic axes, shoot apices and Leaf primordia), owing to their higher competence for regeneration (Molvig et al., 1997; Pigeaire et al., 1997; Babaoglu et al., 2000; Atkins and Smith, 2003; Polowick et al., 2014; Nguyen et al., 2016b).

A major breakthrough was achieved by Molvig et al. (1997) and Pigeaire et al. (1997), who developed *Agrobacterium tumefaciens*-mediated transformation protocols for *L. angustifolius*, using embryonic axes and shoot apices to recover transgenic plants with stable gene integration. However, transformation efficiencies remained low (≤2.8%) and highly genotype-dependent. The approach was subsequently extended to *L. mutabilis* and *L. luteus* (Babaoglu et al., 2000; Li et al., 2000). In *L. luteus*, Li et al. (2000) employed meristem co-cultivation followed by grafting of transformed shoots onto non-transgenic *L. angustifolius* rootstocks, achieving efficiencies of 0.05–0.75% in the T_1_ generation. Efforts to engineer agronomic traits, including herbicide resistance, demonstrated the practical potential of transformation. Atkins and Smith (2003) generated stable, herbicide-tolerant *L. angustifolius* lines with transformation efficiency of ~0.4%. Transgene inheritance in T1 seeds followed a Mendelian 3:1 segregation ratio, affirming stable integration. In contrast, Barker et al. (2016) reported deviations from Mendelian patterns, suggesting persistent chimerism reflecting challenges in achieving uniform transgene integration.

Selection strategies and marker genes have played a critical role in the success of plant transformation. While phosphinothricin (PPT) selection was widely used, Nguyen et al. (2016b) demonstrated that hygromycin selection significantly outperformed standard PPT/*bar* systems in generating transgenic shoots.

Reporter genes such as *uidA* (GUS), *eGFP (enhanced Green Fluorescent Protein*) and *nptII* (*neomycin phosphotransferase II*) have been effectively employed across studies to confirm transformation events. Notably, Pniewski et al. (2006) reported a 44% transformation efficiency in *L. luteus* callus using *nptII* and *uidA* and successfully expressed the Hepatitis B surface antigen (S-HBsAg), illustrating the platform’s utility for recombinant protein production.

Substantial improvements were reported by Pniewski et al. (2006) and Polowick et al. (2014), who optimized culture conditions to raise transformation efficiency significantly. However, these gains were largely restricted to callus induction, with limited whole-plant regeneration and poor reproducibility across species. The most notable advancement came from Nguyen et al. (2016a); Nguyen et al. (2016b), who achieved up to 75% transformation efficiency in *L. angustifolius* through strategic tissue targeting and delayed selection, significantly reducing chimerism. Despite this, the regenerative capacity of transformed tissues remained limited, requiring further subculturing to obtain uniform, heritable transgenic lines.

In parallel to these efforts, *A. rhizogenes*-mediated transformation systems have emerged as powerful tools for functional studies in root biology and nutrient stress adaptation. The initial demonstration by Mugnier (1988), followed by Berlin et al. (1991), confirmed the feasibility of *Agrobacterium rhizogenes*-mediated gene delivery in lupins, resulting in the formation of hairy roots. These studies laid the foundation for gene functional analysis in this genus. However, the inability to regenerate whole plants from transformed tissues remains a key limitation. More recently, the application of hairy root transformation has enabled efficient gene validation. Xu et al. (2020); Aslam et al. (2021) and Aslam et al. (2023) used hairy root systems to investigate gene functions related to phosphorus uptake and root development. These studies enabled rapid gene validation through overexpression of candidate genes such as *PAP10, PAP12* (*Purple acid phosphatase*), *LalbABCG29* (*L. albus ATP-Binding Cassette G family transporter 29*) and *LaGRAS* (*L. albus* GRAS = named after GAI, RGA and SCR) family members, thereby contributing to a mechanistic understanding of abiotic stress resilience in lupins.

### 3.2 Biolistic based transformation

The development of particle bombardment has emerged as an effective alternative for delivering DNA into plant cells, particularly in species resistant to *Agrobacterium*-mediated transformation (Ozyigit and Yucebilgili Kurtoglu, 2020). This biolistic method enables the direct transfer of nucleic acids or ribonucleoprotein (RNP) complexes by coating them onto gold or tungsten particles, which are then accelerated via high-pressure helium discharge to penetrate physical barriers and deliver genetic material into key organelles such as the nucleus and chloroplast. Notably, biolistic transformation facilitates genome editing without T-DNA integration while delivering high gene dosages (Eudes et al., 2014; Ozyigit and Yucebilgili Kurtoglu, 2020).

Particle bombardment-based transformation has been successfully applied in various legume species, demonstrating its versatility across explant types. In soybean, embryonic axes were effectively transformed (Aragão et al., 2000; Rech et al., 2008), while cowpea studies utilized embryonic axes (Ivo et al., 2008; Cruz and Aragão, 2014; Grazziotin et al., 2020). Similarly, chickpea transformation was achieved using epicotyls and embryonal axes (Indurker et al., 2007), whereas pigeon pea relied on cotyledonary nodes (Thu et al., 2007) and leaf explants (Dayal et al., 2003). In black gram, cotyledonary nodes were targeted for transformation (Das, 2018) and in alfalfa, calli derived from petioles and stem sections proved amenable to biolistic delivery (Pereira and Erickson, 1995).

Despite these successes in related legumes, biolistic transformation has not yet been reported in lupins. While the shoot apical meristem remains the primary target for *Agrobacterium*-mediated transformation in lupins, the low amenability of this genus to transformation necessitates the exploration of alternative methods, including particle bombardment

### 3.3 Protoplast-based transformation and regeneration

The intrinsic properties of protoplasts, particularly the absence of a rigid cell wall, facilitate high transformation efficiencies. In *L. albus*, Wetten et al. (1999) demonstrated the use of polyethylene glycol (PEG)-mediated transfection for direct gene delivery into protoplasts, establishing a critical proof-of-concept for genetic manipulation in this transformation-challenged legume. Their investigations identified critical factors affecting transformation success, including the molecular weight and concentration of PEG, plasmid DNA levels and magnesium ion concentration. While these findings laid the foundation for optimizing gene delivery protocols, stable transformation and subsequent plant regeneration were not achieved, reflecting a broader limitation across many crop species.

## 4 Genome editing in lupins

Genome editing represents a contemporary and increasingly prevalent application in the domain of crop breeding (Zhang et al., 2018; Chen et al., 2024). Although it has been applied in crop improvement for over a decade, recent advancements have led to the continuous emergence of more refined and versatile editing tools (Capdeville et al., 2023; Chen et al., 2024). These include base editors (Molla et al., 2021), prime editors (Vu et al., 2024), homology-directed repair (Schreiber et al., 2024), micro-homology-mediated end joining (Van Vu et al., 2021) and chromosome engineering (Puchta and Houben, 2024). The deployment of these tools has enabled the development of crops with enhanced nutritional quality (Kumar et al., 2022), increased pathogen resistance (Pathi et al., 2020; Schenke and Cai, 2020; Pathi, 2021) and enhanced adaptability to changing environments (Chennakesavulu et al., 2021).

In legumes, genome editing offers a versatile approach to improving traits beyond stress resistance, including the removal of allergenic or anti-nutritional compounds (e.g., in peanut and grass pea) (Xu et al., 2018; Biswas et al., 2022; Verma et al., 2023) and the functional dissection of genes related to symbiotic nitrogen fixation (Wang et al., 2017, 2019). Despite these advantages, genome editing in legumes remains significantly hindered by low transformation efficiency, which restricts the production of edited events necessary for downstream selection.

To circumvent this challenge, transient protoplast assays have emerged as a valuable alternative in species where stable transformation is inefficient. These assays allow for the rapid pre-screening of guide RNAs (gRNAs) by testing their cleavage efficiency in isolated cells, thereby enabling the selection of high-performing gRNAs prior to stable transformation. Such strategies have been successfully applied in *Vigna unguiculata* (Bridgeland et al., 2023), *Arachis hypogaea* (Yuan et al., 2019; Biswas et al., 2022), and *Cicer arietinum* (Badhan et al., 2021), facilitating targeted mutagenesis in these species.

Among legumes, soybean remains the most advanced model for genome editing due to the availability of reliable transformation systems (Li et al., 2015). Genome editing has been used to improve yield-related traits such as node length and pod number (Chen et al., 2020), alter flowering time (Cai et al., 2018, 2020) and enhance amino acid content (Do et al., 2019). Notably, high-oleic acid soybeans became the first gene-edited crop to reach commercial markets in the United States (Ledford, 2016). Although editing in other legumes has been attempted with good editing efficiencies in the T₀ generation, these are tissue-specific rather than regenerated genome-edited plants (Juranić et al., 2020; Gupta et al., 2023). Nonetheless, examples of regenerated, edited plants include yellow pea lines with improved flavor and fatty acid profiles (Bhowmik et al., 2023) and alfalfa lines with enhanced yield through altered leaf-to-stem ratios (Zhao et al., 2024).

Recent advances in CRISPR-based genome editing have substantially enhanced functional genomics in *L. albus*, particularly for traits related to nutrient uptake and abiotic stress tolerance. The successful editing of the *MATE* (*Multidrug and Toxic Compound Extrusion*) and *ALMT* (*Aluminum-Activated Malate Transporter*) genes, critical for aluminum toxicity tolerance (Zhou et al., 2020, 2021), highlights the potential of genome editing for improving environmental resilience in lupin. The optimization of multiplex genome editing through *A. rhizogenes*-mediated transformation further broadened this toolkit, enabling simultaneous targeting of multiple genes, as demonstrated with the *Lalb_Chr05g0223881* trehalase gene (Zhu et al., 2023). However, despite these advances, *A. rhizogenes*-mediated transformation remains restricted to root tissues full-plant regeneration remains the bottleneck, limiting the evaluation of whole-plant traits essential for comprehensive crop improvement.

## 5 Potential challenges

Multiple interdependent factors govern the efficiency of *in vitro* plant regeneration and genetic transformation in *Lupinus* species, with genotype dependency, explant selection, culture medium composition, *Agrobacterium* strain and selection marker systems representing key determinants. Despite significant research efforts, the low responsiveness of lupins to *in vitro* regeneration and stable genetic transformation remains a major bottleneck, impeding progress in genetic improvement programs. Systematic exploration of various regeneration strategies has yielded limited success, underscoring the need for more efficient and reproducible protocols.

Early attempts to achieve lupin regeneration through somatic embryogenesis, particularly from immature cotyledons, have been largely unsuccessful, with low regeneration efficiency reported primarily in *L. albus* (Nadolska-Orczyk, 1992; Rybczyitski and Podyma, 1993). Organogenesis-based regeneration from meristematic tissues has also been investigated; however, transformation attempts using these tissues often result in chimeric plants, with transformation efficiencies remaining exceptionally low. Similarly, biolistic gene gun transformation, a promising alternative, has been scarcely explored in lupins, highlighting a critical gap in the development of robust transformation methodologies.

Protoplast isolation has been successfully achieved in lupins, but progress in subsequent callus formation and whole-plant regeneration has been minimal, representing a significant technical challenge. Double haploid (DH) technology through microspore and anther culture has achieved limited success in lupins, primarily due to low *in vitro* responsiveness, inefficient exine rupture, limited callus formation and poor regeneration rates. These challenges have constrained the effective application of DH technology in lupin breeding programs.

The implementation of precision breeding technologies, including targeted genome editing approaches, remains in the early stages of development for lupins. While a few studies have reported the generation of mutated alleles via *A. rhizogene*s-mediated transformation, these applications remain tissue-specific and have not yet achieved full-plant regeneration. Establishing efficient and reproducible genome editing protocols is essential to unlock the full potential of lupin genetic improvement.

## 6 Future perspectives

Addressing key biological constraints, such as genotype dependence and tissue-specific regeneration limitations in lupins, is critical for advancing automated transformation systems and enhancing their efficiency and scalability. While conventional approaches involving plant growth regulators and nutrient optimization have shown limited success. The challenges posed by genotype dependency can be partially mitigated through fundamental research aimed at elucidating the underlying biological processes and genetic mechanisms. For instance, identifying genes and pathways associated with genotype dependency is crucial. A notable example is the knockout of *SAUR15*, an early auxin-responsive gene in maize, which significantly enhanced regeneration efficiency (Wang et al., 2022). Such insights highlight the potential of targeted genetic modifications to overcome regeneration barriers.

Recent studies demonstrate that the expression of developmental regulators (DRs) can significantly enhance regeneration capacity and transformation efficiency in recalcitrant species (Gordon-Kamm et al., 2019). For instance, the *GRF4–GIF1* chimera (*GROWTH-REGULATING FACTOR4–GRF-INTERACTING FACTOR1*) successfully overcame regeneration and transformation recalcitrance in durum wheat, bread wheat and triticale (Debernardi et al., 2020). Similarly, constitutive expression of *GRF5* in sugar beet accelerated shoot organogenesis and improved transformation efficiency in hard-to-transform varieties (Kong et al., 2020). In addition to GRFs, several other developmental regulators such as *SOMATIC EMBRYOGENESIS RECEPTOR-LIKE KINASE (SERK)*, *WOUND-INDUCED DEDIFFERENTIATION1 (WIND1)*, *LEAFY COTYLEDON1 and 2 (LEC1/2)*, *WUSCHEL (WUS)* and *BABY BOOM (BBM)* have demonstrated significant potential in enhancing regeneration across a variety of plant species by helping to bypass recalcitrance-related barriers (Braybrook and Harada, 2008; Jha and Kumar, 2018; Lian et al., 2022; Yarra and Krysan, 2023; Xu et al., 2024). Targeted expression of DRs offers a potential solution to lupin recalcitrance in regeneration and transformation. Moreover, integrating DR genes into protoplast-based systems could enhance regeneration efficiency, paving the way for improved protoplast fusion and transformation. Successful protoplast regeneration would further enable the use of RNP complexes for precise genome editing, thereby expanding the genetic engineering toolkit for lupin improvement.

*Agrobacterium*-mediated transformation is progressing in multiple directions, with various strategies being developed to address transformation challenges. These include the utilization of mutated versions of virulence genes, such as *VirGN54D* (Mortensen et al., 2019) and the adoption of ternary vector systems incorporating helper plasmids containing additional virulence genes, such as *pSB1*, *pHP71539, pVS1-VIR2 and pKL2299* (Komari et al., 1996; Kumlehn et al., 2006; Anand et al., 2018; Zhang et al., 2019; Kang et al., 2022). Additionally, engineered *Agrobacterium* strains utilizing a type III secretion system to deliver *Pseudomonas* effectors effectively suppressing host defense responses have markedly increased transformation efficiency in crops like wheat, alfalfa and switchgrass (Raman et al., 2022). Further, the use of auxotrophic strains, such as *LBA4404 Thy-* and *EHA105 Met-*, minimizes the need for antibiotics to prevent *Agrobacterium* overgrowth on tissues, thereby aiding in the optimization and streamlining of transformation protocols (Lowe et al., 2018; Prías-Blanco et al., 2022; Zhong et al., 2025b).

Alongside these advancements, the progress in the development of tissue culture-free transformation (TCFT) systems represents a significant breakthrough in plant biotechnology, offering a promising solution to the persistent challenge of genotype dependency and bypass the need for lengthy *in vitro* regeneration (Zhong et al., 2025a). Beyond conventional double haploid production methods, emerging approaches utilizing haploidy inducers are gaining traction as alternative strategies for DH generation (Lv and Kelliher, 2023). These methodologies hold great promise for accelerating breeding programs and facilitating the rapid development of superior lupin cultivars.

Collectively, these biotechnological advancements hold transformative potential for overcoming existing limitations and unlocking novel opportunities for the genetic enhancement of lupins.

## Author’s Note

Author KMP has senior authorship for this article.
